# Molecular characterization of chickpea chlorotic dwarf virus and peanut witches’ broom phytoplasma associated with chickpea stunt disease and identification of new host crops and leafhopper vectors in India

**DOI:** 10.1007/s13205-020-02613-7

**Published:** 2021-02-03

**Authors:** Madem Gurivi Reddy, Virendra Kumar Baranwal, Doddachowdappa Sagar, Govind Pratap Rao

**Affiliations:** 1 Department of Plant Pathology, S.V. Agricultural College, Tirupati, Andhra Pradesh 517502 India; 2 Division of Plant Pathology, Indian Agriculture Research Institute, Pusa Campus, New Delhi, 110012 India; 3 Division of Entomology, Indian Agriculture Research Institute, Pusa Campus, New Delhi, 110012 India

**Keywords:** Multilocus gene, PCR assay, *Cicer arietinum*, Mastrevirus, 16SrII-C, 16SrII-D Phytoplasma subgroups, Leafhopper vector

## Abstract

An investigation was carried out to identify and characterize the phytoplasma and viruses associated with the chickpea varieties showing severe stunting, leaf reddening, yellowing and phyllody symptoms during the summer season of 2018–2019 and 2019–2020 in eight states of India. The average disease incidence was recorded from 3 to 32% in different states. The presence of chickpea chlorotic dwarf virus (CpCDV) was confirmed in thirty-seven chickpea samples by amplification of CpCDV coat protein gene and sequence comparison analysis. No record of association of luteovirus, polerovirus and cucumovirus could be detected in any of the symptomatic chickpea samples by RT-PCR assay. *Brassica nigra*, *B. juncea, Lens culinaris*, two weeds (*Heteropogan contartus, Aeschynomene virginica*) and one leafhopper (*Amarasca biguttula*) were identified as new putative hosts for CpCDV. Association of peanut witches’ broom phytoplasma was confirmed in twenty-eight chickpea samples, *Sesamum indicum*, five weeds hosts and two leafhopper species (*Exitianus indicus, Empoasca motti*) using nested PCR assays with primer pairs P1/P7 and R16F2n/R16Rn. The results of phytoplasma association in plants and leafhopper samples were further validated by using five multilocus genes (*sec*A, *rp, imp, tuf* and *sec*Y) specific primers. Sequence comparison, phylogenetic and virtual RFLP analysis of 16S rRNA gene and five multilocus genes confirmed the identity of association of 16SrII-C and 16SrII-D subgroups of phytoplasmas strain with chickpea samples collected from Andhra Pradesh (AP), Telangana, Karnataka, Madhya Pradesh, Uttar Pradesh and New Delhi. Mixed infection of phytoplasma (16SrII-D) and CpCDV was also detected in symptomatic chickpea samples from AP and Telangana. The reports of association of 16SrII-C subgroup phytoplasma in chickpea and 16SrII-D subgroup phytoplasma in *C. sparsiflora* and *C. roseus* are the new host records in world and from India, respectively.

## Introduction

Chickpea (*Cicer arietinum* L.), known as bengal gram, belongs to family fabaceae and is cultivated in more than 50 countries of Asia, Europe, Australia, North America and South America. The highest productivity of 4,770.82 kg/ha is recorded in Israel followed by China, Uzbekistan Yemen and Egypt, whereas India’s average productivity is only 935.34 kg/ha (Merga and Haji 2019). In India, chickpea is grown almost in all parts of the country mainly as a rainfed crop (68% area). During 2018–2019, chickpea production in India has been estimated to be about 10.09 million tons, which is about 43% of the total pulse production (23.22 mt) in India. Madhya Pradesh (MP), Rajasthan, Maharashtra, Uttar Pradesh (UP), Andhra Pradesh (AP), Karnataka, Chhattisgarh, Bihar and Jharkhand states contribute more than 95% of the total chickpea production in the country. The area, production and productivity of chickpea in India have been growing at significant rate during the past decade (Anonymous 2019).

Chickpea is affected by many biotic stresses (ascochyta blight, botrytis gray mold, dry rot, phyllody, stunt, wilt, etc.) and abiotic stresses (cold, drought, heat, salinity, etc.) (Singh et al. 1993). It is estimated that chickpea suffers on an average loss of 25–30% due to various biotic and abiotic stresses. Among major biotic stresses, chickpea stunt is a leading disease caused by different groups of viruses, e.g., cucumovirus, mastrevirus and polerovirus, and is reported in many chickpea growing countries worldwide (Akram et al. 2016; Abraham et al. 2006; Chalam et al. 1986; Kanakala et al. 2013; Kanakala and Kuria 2019). The major symptoms reported by different workers associated with stunt disease include leaf reddening and stunting in desi-type and leaf yellowing in kabuli-type along with browning of vascular tissues in collar regions. In early stage of the crop, diseased plants show more severe stunting symptoms (Nene and Reddy^1987^; Kanakala and Kuria 2019; Shreenath et al. 2020).

Chickpea stunt disease (CpSD) was first documented in Iran and showed the association of bean leaf roll virus (BLRV) and chickpea chlorotic dwarf virus (CpCDV) (Kaiser 1972). Later, CpCDV was confirmed and characterized with spring and summer CpS disease in Syria (Kumari et al. 2004) and in India (Reddy et al. 1979; Horn et al. 1993). Different luteoviruses were also reported to cause stunt disease in chickpea worldwide, for example, subterranean clover red leaf virus (SCRLV), beet western yellows virus (BWYV) in California (Bosque-Perez and Buddenhagen 1990; Horn et al. 1993) and BLRV and BWYV in Spain (Carazo et al. 1993). A new chickpea chlorotic stunt virus (CpCSV) strain of the genus *Polerovirus* was identified to infect chickpea in Ethiopia causing yellowing and stunting symptoms (Abraham et al. 2006).

Although the different groups of viruses are identified as a causal pathogen of CpSD worldwide, CpCDV was recognized as the major virus prevailing across the Indian subcontinent, Middle East and North Africa (Horn et al. 1996; Kanakala and Kuria 2019). CpCDV was reported to be transmitted by *Orosius orientalis* and *O*. *albicinctus* (Horn et al. 1993, 1996; Kumari et al. 2004). CpCDV was later reported to affect chickpea and other legume crops in Australia, Egypt, Iraq, Iran, Oman, Pakistan, Sudan, Syria and Yemen (Kanakala and Kuria 2019). Although CpCDV has been reported as a major virus associated with CpSD, the presence of luteovirus and cucumovirus is also documented from India (Kanakala et al. 2013; Shreenath et al. 2020).

Phytoplasma association has also been described with CpSD. Chickpea phyllody disease is reported to be caused by 16SrII-D subgroup phytoplasma strain from Pakistan, Oman, Australia, Myanmar and Sudan (Akhtar et al. 2008; Al-Saady et al. 2006; Saqib et al. 2005; Reddy et al. 1991). In India, phytoplasma association with chickpea phyllody was first reported from Tamil Nadu state of India (Venkataraman 1959; Kandaswamy and Natarajan 1974). Average yearly estimated yield loss of ~ 15% was reported in chickpea varieties due to phyllody diseases in India (Ghanekar et al. 1988). The phytoplasma strain associated with chickpea phyllody was reported to be transmitted by *O. albicinctus* in Pakistan (Akhtar et al. 2009). The incidence of phytoplasma associated with chickpea has been reported increasing throughout major chickpea growing areas of India (Pallavi et al. 2012; Akram et al. 2016; Shreenath et al. 2020).

Consistent occurrence of chickpea wilt and stunt disease causes serious yield loss to the chickpea crop every year in all major chickpea growing areas of India (Malathi and Kanakala 2017). But no detailed study has been performed to record the CpSD incidence in major chickpea production areas of India along with the identity of pathogen(s) involved. In the present investigation, an attempt was made to investigate the distribution, occurrence and pathogens associated with chickpea stunt disease in eight states of India along with the possible sources of their natural spread.

## Material and methods

### Survey and collection of plant samples

A roving survey was conducted in farmer’s fields and chickpea experimental plots at ICAR Research centers and Agriculture University campuses in eight chickpea growing states of India (AP, Telangana, Karnataka, MP, Gujarat, Rajasthan, UP and New Delhi) during summer season of 2018–2019 and in three states (AP, Telangana and New Delhi) during summer season of 2019–2020. In total, 12 districts in 8 states were surveyed which included two districts each in AP (Kadapa, Kurnool), UP (Kanpur, Meerut), MP (Indore, Jabalpur), Rajasthan (Udaipur, Ganganagar), and one district each in Telangana (Ranga Reddy), Karnataka (Dharwad) and New Delhi (IARI, Research fields) for chickpea stunt and phyllody disease. In each chickpea field, a plot area of 5 × 5 m was selected and the total number of healthy and symptomatic plants showing leaf reddening, stunt and phyllody symptoms was recorded and the percent disease incidence was calculated. Three spots were randomly selected in each field, and the mean of disease incidence was calculated in individual field/experimental plots.

The per cent disease incidence was calculated by averaging the incidence of three spots at each location by using the formula.

$${\text{Percent disease incidence}} = \frac{{{\text{No}}.{\text{ of plants infected}}}}{{{\text{Total no}}{\text{. of plants}}}} \times 100$$

The overall average disease incidence in a particular district was calculated by taking the mean of disease incidence calculated in all the fields.

Symptomatic chickpea plants were collected from different survey locations. Weeds and nearby growing crops in and adjoining chickpea fields showing suspected virus and/or phytoplasma symptoms were also collected. Non-symptomatic chickpea, other crops and weeds samples were also collected from each surveyed locations, which were used as PCR negative controls. All the weed species were taxonomically identified from Agronomy Division of IARI, New Delhi. All the collected plant samples were packed in polythene bags and kept in deep freezer at − 80 °C for PCR/RT-PCR analysis.

### Collection and identification of insects

The leafhopper and aphid species feeding on chickpea plants of surveyed fields were collected from AP and New Delhi using yellow sticky traps and sweeping nets. Collected leafhoppers/aphids were carefully stored in plastic vials at 4 °C in 70% ethanol for further identification and PCR analysis. The collected insects were submitted to Division of Entomology, IARI, New Delhi, for identification.

### Detection of CpSD-associated viruses

#### DNA isolation from plant and insect samples

Total genomic DNA was isolated from the symptomatic and asymptomatic plant samples using Qiagen DNeasy plant mini kit (Germany) as well as from the whole body of insects using Qiagen blood tissue kit (Germany) as per manufacturer protocol from different surveyed locations and used as a template for PCR assays.

#### Identification of leafhopper species by PCR assay using *COX1* gene

For the identification of leaf hopper species, PCR amplification of partial mitochondrial *COX* I gene (Cytochrome Oxidase I, COI) was performed using HCO and LCO primer pairs (Folmer et al. 1994). The major leafhoppers/plant hoppers/aphid species identified in the study collected from AP and New Delhi were analyzed for presence of virus/phytoplasma by specific PCR/RT-PCR assays.

#### Identification of DNA viruses (mastrevirus) by PCR assays

A set of partial coat protein (CP) specific primer pair (MCPF/MCPR) was used to identify the CpCDV (Kanakala et al. 2013).

#### Identification of RNA viruses (luteoviruses, poleroviruses and cucumovirus) associated RT-PCR assays

##### RNA isolation from plant samples

RNA was isolated from symptomatic and non-symptomatic leaf samples of chickpea and other plants species by using QIAGEN RNeasy Plant Mini Kit as per manufacturer description.

##### RT-PCR assay

### Reverse Transcription PCR (RT-PCR) assays for cucumovirus, luteovirus and polerovirus detection

cDNA synthesis was performed by using a Verso cDNA synthesis Kit (Thermo scientific). The cDNA was used for PCR amplifications of BLRV using the primer pair BLRV-3/ BLRV-5 (Ortiz et al. 2005), cucumber mosaic virus (CMV) using the primer pair CPF/CPR (Shreenath et al. 2020) and Lu1 + Eco side/Lu4 + Eco primer pair for the plant viruses belonging to the family *Luteoviridae* (Robertson et al. 1991).

#### Identification of phytoplasma by PCR assays

The extracted DNA was amplified for 16S ribosomal DNA with phytoplasma specific universal primer pair P1/P7 (Deng and Hiruki 1991; Schneider et al. 1995) followed by nested primer pair R16F2n/R16R2 (Gundersen and Lee 1996) from the plants and insects.

Amplification of five multilocus candidate genes (*sec*A, *rp*, *sec*Y, *imp* and *tuf*) were employed by the utilization of primer pairs: *sec*A (SecAfor1/SecArev3 followed by nested PCR primers SecAfor5/ SecArev2) (Hodgetts et al. 2008; Bekele et al. 2011)*, rp* (rp(II)F/rp(I)R1A followed by semi-nested PCR primers rp(II)F2/ rp(I)R1A) (Martini, 2004), *sec*Y (SecYF1(II)/SecYR1(II) followed by semi-nested PCR primers SecYF2 (II)/SecYR1 (II)) (Lee et al. 2010), *tuf* genes (EF-Tu) (TUF-II-F1/TUF-II-R1 followed by semi-nested PCR primers TUF-II-F2/TUF-II-R1) and *imp* gene (IMP-II-F1/IMP-II-R1 followed by semi-nested PCR primers IMP-II-F2/IMP-II-R1) (Al-Subhi et al. 2018).

### Nucleotide sequencing

At least two recombinant clones and/or direct PCR amplified products were sequenced directly in both directions using the same set of primers as for the PCR amplification at Eurofins Genomics Pvt., Ltd., Karnataka. The pair-wise sequence comparison analysis was done through BLAST analysis. The original forward and reverse sequence data of each test samples were edited, aligned and assembled with CLC Genomics Workbench 12.0 (https://www.qiagenbioinformatics.com/) and sequences of the representative strains were deposited in GenBank (NCBI, Bethesda, MD, USA) data library and accession numbers were received.

### Phylogenetic analysis

Nucleotide sequences of different representative virus and phytoplasma groups were retrieved from GenBank and were aligned with virus and phytoplasma sequences available in GenBank using CLC Genomics Workbench 12.0 (https:// www. qiagen bioinformatics.com). Phylogenetic trees were constructed using the neighbor-joining method for phytoplasma and maximum likelihood for virus with MEGA 6.0 software (Kumar et al. 2016) using 1000 bootstrap replications. Sequences of tomato leaf curl Palampur virus (ToLCPalV) were used as an outgroup to root the phylogenetic trees of CpCDV partial coat protein gene, whereas *Acholeplasma laidlawii* (Acc. no. AB680603) was used as an outgroup to root the phylogenetic trees of 16S ribosomal gene and *Bacillus subtilis* (Acc. no. BALZ01000186) to root the phylogenetic trees of the *sec*A, *sec*Y and *tuf* genes.

### Virtual RFLP analysis

Virtual RFLP analysis was carried out for R16F2n/R16R2 fragments of 16Sr RNA gene derived from identified phytoplasma strains from plants and insects and was submitted to *iPhy*Classifier online tool (Zhao et al. 2009). The different restriction profiles, obtained with 17 restriction endonucleases (*Bam*HI, *Bfa*I, *Alu*I, *Bst*UI, *Hae*III, *Eco*RI, *Dra*I, *Hin*fI, *Hpa*II, *Hha*I, *HpaI*, *Kpn*I, *Sau3*AI, *Ssp*I, *Rsa*I, *Mse*I, *Taq*I) of different phytoplasma isolates in virtual gel plotting, were compared with the virtual RFLP pattern from the standard representative group/subgroup reference strains of phytoplasma by the same restriction enzymes and similarity coefficient values.

## Results

### Survey, disease incidence and symptomatology

Roving survey of chickpea fields in eight states of India during 2018–2019 summer season revealed a wide spread occurrence of chickpea stunt and leaf reddening disease in all the states. Association of phyllody and witches’ broom symptoms was also observed in the states of AP, Karnataka, Telangana, MP, UP and New Delhi (Table 1).

**Table 1:** Survey, symptoms and percent disease incidence of chickpea stunt and phyllody disease from different states of India during 2018–2020

State	District	Location	*No. of fields	Year	Symptoms	Disease Incidence	
						Range	Average**
Andhra Pradesh	Kadapa	Farmer fields	16	2018–2019	Leaf reddening, phyllody, stunting, leaf rolling, little leaf, and yellowing	5–35	16
	Kurnool	Farmer fields	8		Leaf reddening, phyllody and stunting	4–38	20
		RARS, Nandyal	5		Leaf reddening, yellowing and phyllody	2–60	15
	Kadapa	Farmer fields	12	2019–2020	Leaf reddening, phyllody, stunting, leaf rolling, little leaf, and yellowing	2–16	6
	Kurnool	Farmer fields	7		Leaf reddening, phyllody, stunting, leaf rolling, little leaf, and yellowing	4–24	13
		RARS, Nandyal	5		Leaf reddening, phyllody, stunting, leaf rolling, little leaf, and yellowing	0–11	3
Telangana	Ranga Reddy	Farmer fields	10	2018–2019	Phyllody, proliferation of axillary shoots, bushy appearance, stunting, leaf yellowing and reddening	2–44	18
		ICRISAT	7		Phyllody, reddening and leaf yellowing	9–23	22
	Ranga Reddy	Farmer fields	10	2019–2020	Phyllody, reddening and leaf yellowing	0–27	11
		ICRISAT	4		Stunting, reddening and leaf yellowing	5–6	5
Karnataka	Dharwad	UAS, Dharwad	3	2018–2019	Phyllody, reddening and stunting	5–8	7
Madhya Pradesh	Indore	Farmer fields	6	2018–2019	Phyllody, yellow orange decoloration and stunting	0–7	3
	Jabalpur	Farmer fields	5		Stunting, reddening and yellow decoloration of leaves	5–7	5
		JNKVV	3		Stunting and reddening	5–8	7
Gujarat	Junagadh	Farmer fields	3	2018–2019	Reddening and phloem discoloration at the collar region	33–38	32
		JAU	3		Reddening and phloem discoloration at the collar region	4–28	18
Rajasthan	Ganganagar	Farmer fields	3	2018–2019	Stunting, typical reddening and orange yellow discoloration	4–20	10
		ARS	2		Stunting, typical reddening and orange yellow discoloration	7–23	15
	Udaipur	Farmer fields	4		Stunting, typical reddening and orange yellow discoloration	0–5	3
Uttar Pradesh	Kanpur	Farmer fields	3	2018–2019	Phyllody, stunting and typical reddening	3–8	6
		CSA	1		Stunting and typical reddening	28	28
		IIPR	1		Stunting and typical reddening	4	4
	Meerut	SVPUAT	1		Phyllody, stunting and typical reddening	8	8
New Delhi	New Delhi	Research plots	3	2018–2019	Stunting, phyllody, yellowing and reddening	9–26	17
			4	2019–2020	Stunting, phyllody, yellowing and reddening	7–24	13

* Average size of field surveyed at different states was ~ 0.5 ha

** Average incidence was calculated by the calculating the means of incidence in different surveyed fields

**Fig. 1:**
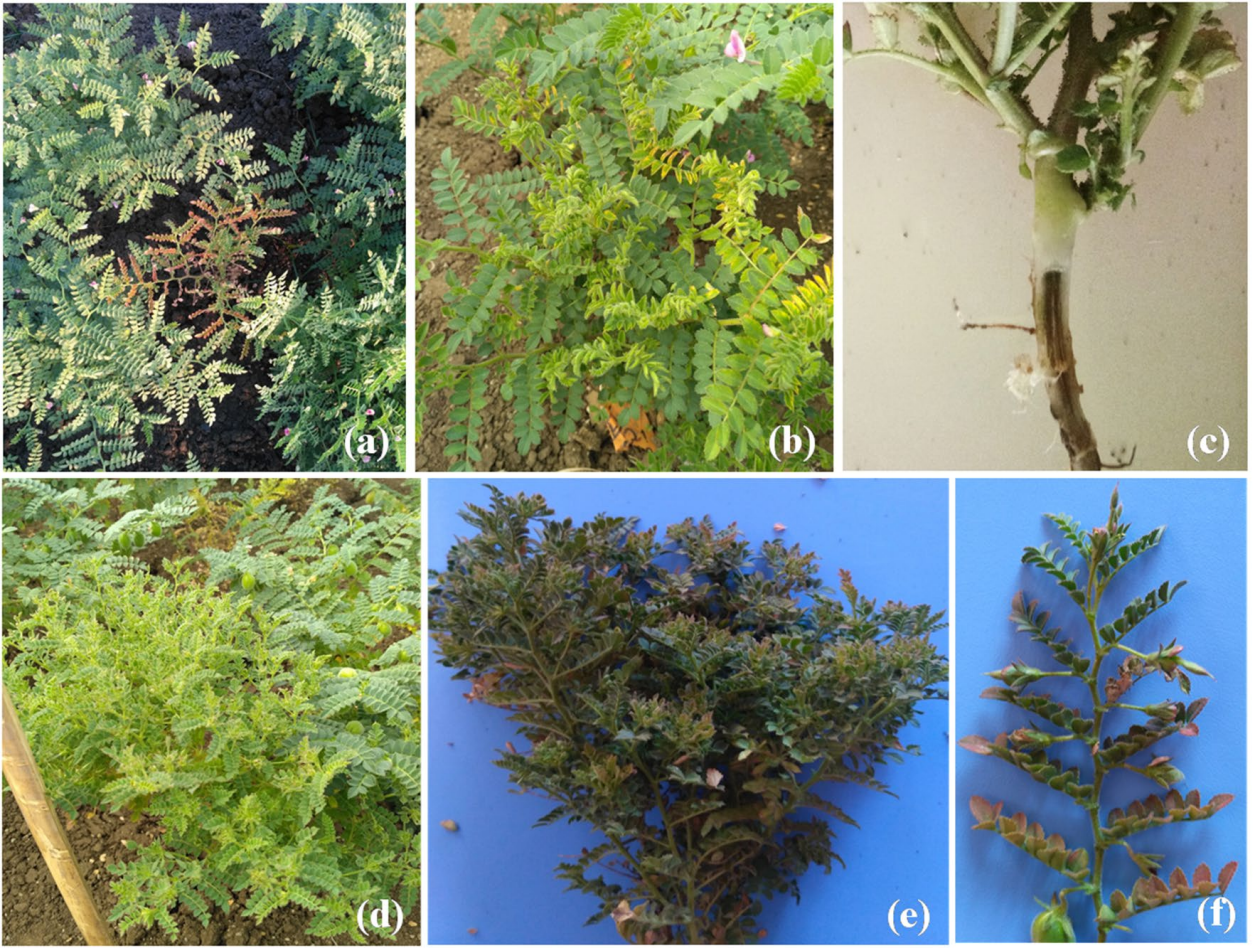
Symptoms of chickpea stunt and phyllody disease in AP: **a** stunting and reddening, **b** stunting, yellowing and leaf rolling, **c** phloem browning at the collar region, **d** phyllody, stunting and yellowing, **e** stunting, phyllody, reddening and bushy appearance, **f** close view of phyllody and reddening symptom

Virus-suspected symptoms of stunting, leaf reddening, phloem discoloration, yellowing and leaf rolling (Fig. 1a–c) and phytoplasma-suspected symptoms of stunting, proliferation of axillary shoots, phyllody and leaf yellowing (Fig. 1d–f) were observed with average disease incidence ranging from 3 to 32% in different chickpea fields of eight states. Subsequently, similar symptoms were also recorded in other surveyed chickpea fields of AP, Telangana and New Delhi during 2019–2020 summer season. But the recorded average disease incidence (3–13%) was lower as compared to the first year (Table 1).

**Fig. 2:**
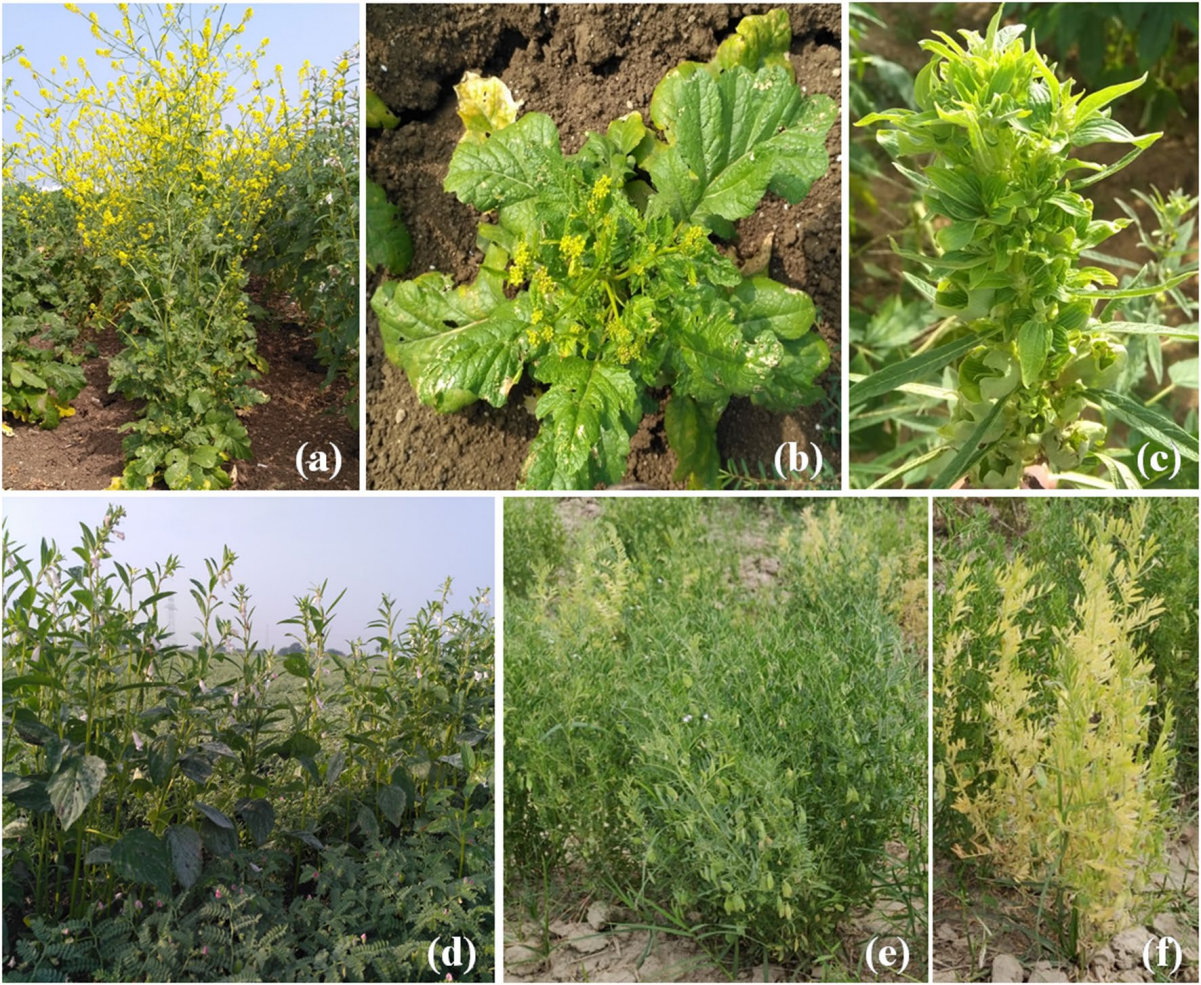
**a** Healthy brassica plant, **b** stunting, yellowing and leaf crinkling symptom in *Brassica nigra*, **c** Sesamum twig showing severe phyllody symptom, **d** healthy *Sesamum indicum* crop grown as inter-crop in chickpea, **e** healthy lentil crop, **f** yellowing and stunting of lentil

Besides chickpea, severe stunting, yellowing and leaf crinkling symptoms were observed in *Brassica nigra* (Fig. 2b) grown nearby chickpea fields at Kurnool district, AP, in 2019–2020. Phyllody symptoms was observed on sesamum plants (Fig. 2c) grown as inter crop in chickpea fields in Kadapa district of AP. *B. juncea* and *Lens culinaris* plants (Fig. 2f) grown nearby chickpea fields were recorded with stunting and bright yellow color symptoms at Kanpur, UP.

**Fig. 3:**
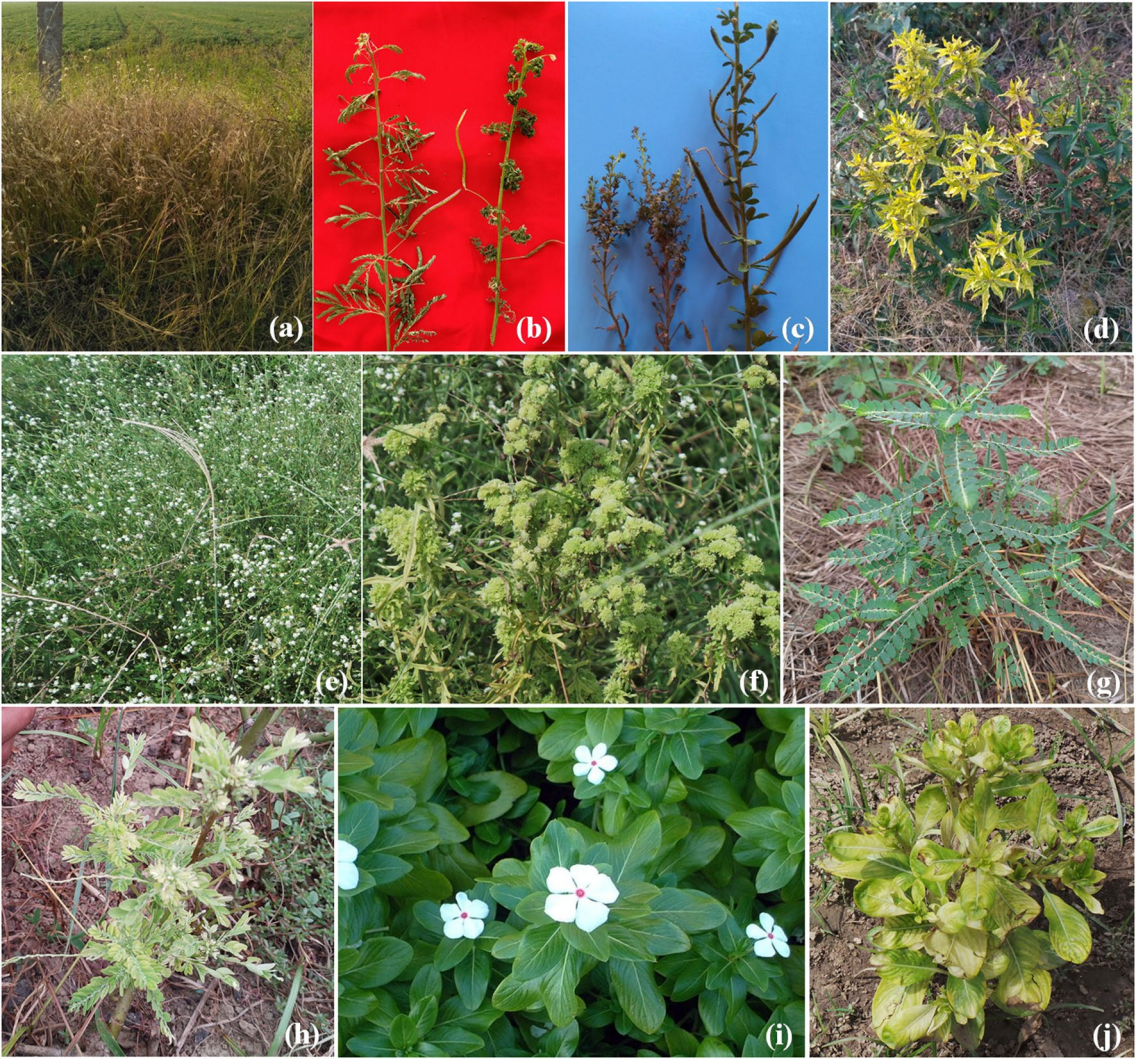
**a** Yellowing and browning of *Heteropogan contartus* plants nearby chickpea fields, **b** leaf crinkling in *Aeshynominae virginia*, healthy twig is on left, **c** witches’ broom symptom on *Cleome viscosa*, healthy twig on right, **d** yellowing of leaves in *Croton sparsiflora*, **e** healthy *Parthenium hysterophorus*, **f** witches’ broom symptom on *Parthenium hysterophorus*, **g** healthy *Phyllanthus niruri*, **h** stunting and witches’ broom symptom in *Phyllanthus niruri*, **i** healthy *Catharanthus roseus*, **j** stunting and yellowing symptom in *Catharanthus roseus*

Symptoms of leaf yellowing were recorded in *Croton sparsiflora* and *Heteropogan contartus*, witches’ broom in *Cleome viscosa* and leaf crinkling in *Aeschynomene virginica* weeds near chickpea fields in AP (Fig. 3a–d). Witches’ broom, stunting and leaf yellowing were observed on *Parthenium hysterophorus* in chickpea fields at Telangana and UP (Fig. 3f). Further phytoplasma symptoms of leaf yellowing and stunting on *Catharanthus roseus* and witches’ broom on *Phyllanthus niruri* (Fig. 3e, g) were recorded nearby chickpea experimental fields at IARI, New Delhi.

### Identification of insect vectors by sequencing of *COX1* gene

The expected DNA fragment of ~ 658 bp was amplified from *COX1* gene in the collected leafhoppers and aphid species (data not shown). Sequencing and BLASTn analysis identified four leafhopper species (*Empoasca motti, Amarasca biguttula* and *Orosius albicinctus* from New Delhi; *Exitianus indicus* from AP) and one aphid species (*Aphis craccivora*) from AP. The sequences of the representative insect vector species were edited and deposited in GenBank.

### Molecular detection of chickpea chlorotic dwarf virus (CpCDV)

An expected amplicon size of ~ 596 bp was consistently obtained by using coat protein gene specific primers of CpCDV in thirty-seven symptomatic chickpea samples (from eight states), *B. nigra* (two samples each from Regional Agricultural Research Station, [RARS], Nandyal, AP), *B. juncea* and *L. culinaris* (two samples each from Kanpur, UP) and two symptomatic weed species, viz. *H. contartus* at Kadapa district of AP and *A. virginica* from RARS, Nandyal, Kurnool district of AP.

Out of four leaf hoppers, only two leafhopper species (*A. biguttula* and *O. albicinctus*) were tested positive in PCR assay by utilizing similar set of CpCDV coat protein specific primer MCPF/R.

The representative sample sequences of chickpea, brassica, lentil and weed species sequences were deposited in GenBank (Table 2).

**Table 2:** Identification of CpCDV, phytoplasma and both from chickpea, other crop hosts, weeds and leafhoppers with different gene specific primers from eight states of India

Crop	State	Year	District	Isolate	Accession Nos						
					Primers of genes utilized for identification of CpCDV and phytoplasma						
					CpCDV	Phytoplasma					
					CP	*16Sr RNA*	*rp*	*sec*A	*sec*Y	*tuf*	*imp*
Chickpea	Andhra Pradesh	2019	Kadapa	CPV-APK2	MT349402	Negative					
				CPV-APK3	MT349403						
				CPAP-P17	Negative	MN551486	MN728264	MN728246	MN728228	MN634232	MN634214
				CPAP-P19		MN551487	MN728265	MN728247	MN728229	MN634233	MN634215
				CPAP-PP2		MN551488	MN728270	MN728252	MN728234	MN634238	MN634220
				CPAP-PP6		MN551489	MN728271	MN728253	MN728235	MN634239	MN634221
				CPAP-J1	MN643059	MN551484	MN728262	MN728244	MN728226	MN634230	MN634212
			Kurnool	CPV-APN4	MT349398	Negative					
				CPV-APN7	MT349399						
				CPAP-N1	Negative	MN551482	MN728260	MN728242	MN728224	MN634228	MN634210
				CPAP-N2		MN551483	MN728261	MN728243	MN728225	MN634229	MN634211
		2020	Kadapa	CPV-P5	MT339206	Negative					
				CPV-P12	MT339207						
				CPV-P6	MT339209						
				CPV-P13	MT339210						
				CPP-P2	Negative	MT420669	MT423334	MT423355	MT423376	MT423397	MT423418
				CPP-P3		MT420670	MT423335	MT423356	MT423377	MT423398	MT423419
				CPP-P8		MT420257	MT423347	MT423368	MT423389	MT423410	MT423431
			Kurnool	CPV-N1	MT339211	Negative					
				CPV-N4	MT339212						
				CPV-N21	MT339213						
				CPV-N24	MT339214						
				CPPY-N3	Negative	MT420259	MT423332	MT423353	MT423374	MT423395	MT423416
				CPPY-N5		MT420260	MT423333	MT423354	MT423375	MT423396	MT423417
				CPP-N7	MT339217	MT420665	MT423328	MT423349	MT423370	MT423391	MT423412
				CPPR-N8	MT339219	MT420667	MT423330	MT423351	MT423372	MT423393	MT423414
	Telangana	2019	Ranga Reddy	CPV-T3	MT349396	Negative					
				CPV-T8	MT349397						
				CPT-I2	Negative	MN551496	MN728258	MN728240	MN728222	MN634226	MN634208
				CPT-I7		MN551497	MN728259	MN728241	MN728223	MN634227	MN634209
		2020	Ranga Reddy	CPP-T1	Negative	MT420261	MT423336	MT423357	MT423378	MT423399	MT423420
				CPP-T2		MT420262	MT423337	MT423358	MT423379	MT423400	MT423421
				CPV-T4	MT395664	MT420671	MT423338	MT423359	MT423380	MT423401	MT423422
	Karnataka	2019	Dharwad	CPV-K2	MT395670	Negative					
				CPV-K3	MT395671						
				CPK-D9	Negative	MN551494	MN728256	MN728238	MN728220	MN634224	MN634206
				CPK-D19		MN551495	MN728257	MN728239	MN728221	MN634225	MN634207
	Madhya Pradesh	2019	Indore	CPV-MP3	MT349392	Negative					
				CPV-MP6	MT349393						
				CPP-MP4	Negative	MT420673	MT423340	MT423361	MT423382	MT423403	MT423424
			Jabalpur	**CPV-JB3**	MT395668	Negative					
				CPP-JB5	Negative	MT420674	MT423341	MT423362	MT423383	MT423404	MT423425
	Gujarat	2019	Junagadh	CPV-GJ3	MT349400	Negative					
				CPV-GJ13	MT349401						
	Rajasthan		Udaipur	CPV-U1	MT395666	Negative					
				CPV-U5	MT395667						
			Ganganagar	CPV-RG1	MT349404						
				CPV-RG3	MT349405						
	Uttar Pradesh	2019	Kanpur	CPV-UP6	MT349394						
				CPV-UP10	MT349395						
				CPP-UP4	Negative	MT420675	MT423342	MT423363	MT423384	MT423405	MT423426
				CPP-UP7		MT420258	MT423348	MT423369	MT423390	MT423411	MT423432
			Meerut	CPV-M1	MT395662	Negative					
				CPV-M8	MT395663						
				CPP-M3	Negative	MT420676	MT423343	MT423364	MT423385	MT423406	MT423427
	New Delhi	2019	IARI	CPV-ND6	MT395660	Negative					
				CPV-ND8	MT395661						
				CP-ND1	Negative	MN551492	MN728254	MN728236	MN728218	MN634222	MN634204
				CP-ND12		MN551493	MN728255	MN728237	MN728219	MN634223	MN634205
		2020	IARI	CPV-ND1	MT349387	Negative					
				CPV-ND5	MT349388						
Other crop hosts											
*Brassica juncea*	UP	2019	Kanpur	BNV-UP8	MT395659	Negative					
*B. juncea*	New Delhi	2019	IARI	BNV-ND4	MT395658						
*B. nigra*	AP	2020	Kurnool	BNF-N3	MT339215	Negative					
				BNF-N13	MT339216						
Lentil	UP	2019	Kanpur	LV-UP2	MT349390	Negative					
				LV-UP5	MT349391						
Sesamum	AP	2020	Kurnool	SP-AP	Negative	MT420677	MT423344	MT423365	MT423386	MT423407	MT423428
Weed species											
*Heteropogan contartus*	AP	2020	Kurnool	HC-P13	MT339208	Negative					
*Aeschynomene* *virgineca*		2020	Kadapa	AV-N3	MT395669						
*Cleome viscosa*		2019	Kadapa	CVAP-P1	Negative	MN551490	MN728268	MN728250	MN728232	MN634236	MN634218
*Croton sparsiflora*		2019	Kurnool	CSAP-P9		MN551491	MN728269	MN728251	MN728233	MN634237	MN634219
*Parthenium* *hysterophorus*	Telangana	2019	Ranga Reddy	PHT-I9	Negative	MN551498	MN728266	MN728248	MN728230	MN634234	MN634216
	UP	2019	Kanpur	PH-UP	Negative	MT420678	MT423345	MT423366	MT423387	MT423408	MT423429
*Catharanthus roseus*	New Delhi	2019	IARI	CR-ND	Negative	MT420679	MT423346	MT423367	MT423388	MT423409	MT423430
*Phyllanthus* *niruri*		2019	IARI	PN-ND6		MN551499	MN728267	MN728249	MN728231	MN634235	MN634217
Leafhoppers											
*Empoasca motti*	New Delhi	2019	IARI	EmND2	Negative	MT500682	MT501706	MT501708	MT501710	MT501712	MT501704
*Exitianus indicus*	AP	2019	Kurnool	EiAPN6	Negative	MT500683	MT501707	MT501709	MT501711	MT501713	MT501705
*Amarasca (Sundapteryx) biguttula*	New Delhi	2019	IARI	VEG1	MT613320	Negative					
*Orosius albicinctus*	New Delhi	2020	IARI	NDI2	MT613321	Negative					

Virus infection Phytoplasma infection Mixed Infection

#### Sequence analysis

BLASTn analysis of partial CP gene sequences of chickpea isolates (Table 2) from eight states, *B. nigra* (Nandyal, AP), *B. juncea* and *L. culinaris* (Kanpur, UP), *H. contartus* (Kadapa, AP), *A. virginica* (Nandyal, AP), and two leafhopper species *A. biguttula* and *O. albicinctus* (New Delhi), shared 99.3% to 99.83% sequence homology with CpCDV isolates from *Spinacea oleracea* (Acc. No. MF178119), *L. culinaris* (Acc. Nos. LN864703, LN865159, LN865160, LN865162) from Pakistan, *Pisum sativum* (Acc. No. KM229786) from Sudan and *C. arietinum* (Acc. No. MG913384) from India.

#### Phylogenetic tree

**Fig. 4:**
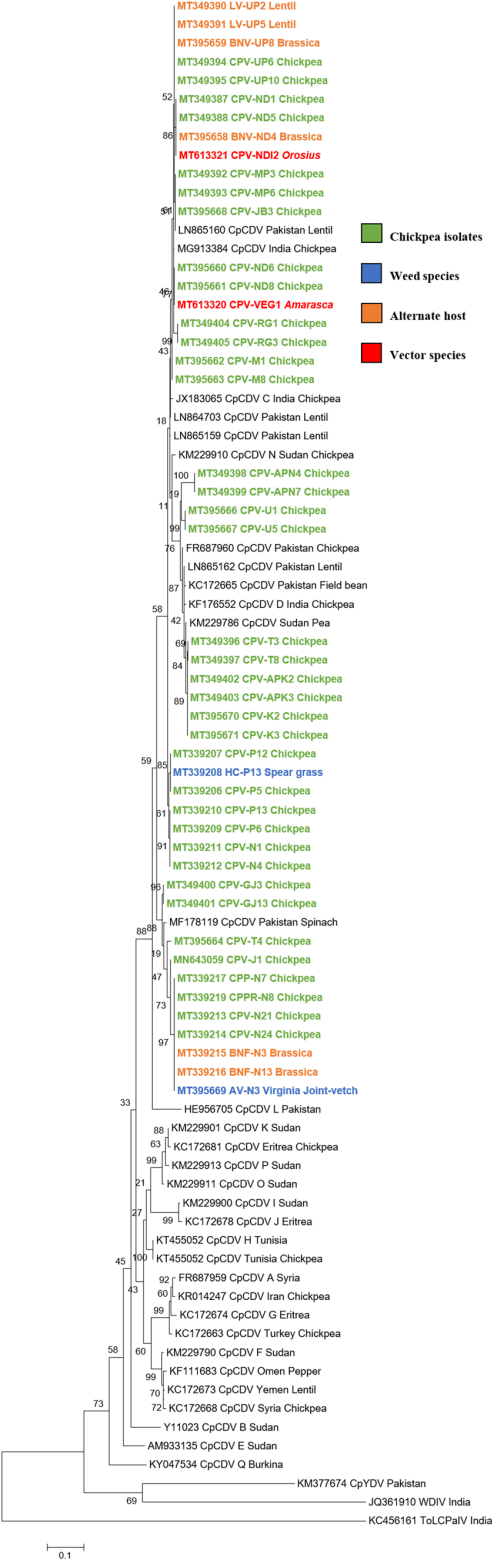
Phylogenetic relationships between dicot infecting mastreviruses. Dendrograms were constructed using maximum likelihood and boot strap (1000 replications) based on alignment of CP sequences of dicot infecting mastreviruses. Alignments were produced with ClustalW. Vertical distances are arbitrary, and horizontal distances are proportional to genetic distances. The numbers at node refer to number of times (as a percentage) in which the branching was supported. The tree was rooted using Tomato leaf curl New Delhi virus (ToLCNDV) as an outgroup

Phylogenetic study based on the coat protein gene sequence of CpCDV isolates associated with naturally infected chickpea plants/other crop hosts/weed species from eight states also suggested that all CpCDV isolates characterized in the study were clustered with CpCDV isolates from lentil, faba bean and spinach from Pakistan, chickpea and pea isolates from Sudan and chickpea isolates reported earlier from India (Fig. 4).

### Detection of RNA viruses associated with symptomatic chickpea samples

Different set of coat protein specific primers were employed to amplify RNA viruses like CpCSV, BLRV and CMV from the chickpea samples collected from eight states of India and *A. craccivora* collected from IARI, New Delhi. No positive amplification was achieved with any of the symptomatic chickpea samples and *A. craccivora* with coat protein specific primers for luteoviruses, BLRV, CpCSV and CMV. The results suggested the absence of association of any of these RNA viruses in the symptomatic chickpea stunt and aphid samples.

### Molecular detection of phytoplasma associated with chickpea

Twenty-eight symptomatic chickpea samples collected from six states of India, viz. AP (Kurnool and Kadapa), Telangana, Karnataka, MP (Indore, Jabalpur), UP (Kanpur, Meerut) and New Delhi (IARI), five weed species, viz. *C. viscosa*, *C. sparsiflora* (AP), *P. hysterophorus* (Telangana and UP), *P. niruri* and *C. roseus* (New Delhi), one other host, viz. *S. indicum* (AP), showing suspected phytoplasma symptoms and the positive control of chickpea phyllody samples yielded ~ 1.8 kb amplified product in first round PCR assays with P1/P7 universal primer pair (data not shown). The positive amplified products of the first round PCR analysis were further processed for nested PCR assays with R16F2n/R16R2 primer pair, which yielded specific amplicons of ~ 1.2 kb from all the symptomatic samples tested in study and also from the positive control of chickpea phyllody phytoplasma isolate (Acc. No. KX151134) maintained in *C. roseus* in the green house **(**data not shown**)**.

However, no DNA amplification was achieved with any of the four identified leafhopper species analyzed in the first-round PCR assays with primer pair P1/P6. In nested PCR analysis, however, ~ 1.2 kb amplified products were obtained from leafhopper *E. indicus,* collected from RARS, Nandyal, AP, and *E. motti* collected from IARI, New Delhi.

No amplifications either in first round or nested PCR assays with similar set of primers were observed in DNAs isolated from any of the plant samples and leaf hoppers (negative control) collected from the distant healthy fields. Nested PCR amplified products were sequenced, and the partial 16S rRNA gene sequences were deposited in the GenBank database (Table 2).

The DNA extracted from the above samples were also analyzed with *rp, secA, secY, tuf* and *imp* gene specific primers. PCR products of ~ 1390 bp and ~ 1290 bp with direct rp(II)F/rp(I)R1A and semi-nested rp(II)F2/rp(I)R1A primers, ~ 840 bp and ~ 600 bp with SecAfor1⁄SecArev3 primer pair followed by SecAfor5⁄SecArev3, ~ 1.7 kb amplicons with direct secYF1(II)/secYR1(II) and semi-nested secYF2(II)/secYR1(II) primer pairs, ~ 1094 bp amplicon size with semi-nested TUF-II-F2/TUF-II-R1 primer and 717 bp with semi-nested IMP-II-F2/IMP-II-R1 primer pairs were consistently amplified in the symptomatic chickpea, sesame, weed species and two leafhoppers (*E. indicus* and *E. motti*) collected from symptomatic chickpea fields. No amplification was achieved with the DNA from the non-symptomatic as well as healthy plant samples neither in first round nor in nested PCR assays with similar set of *sec*A*, rp, sec*Y *tuf* and *imp* gene group specific primers. The multilocus gene PCR products were sequenced, analyzed and deposited in the GenBank database (Table 2).

#### Sequence analysis

Pairwise sequence comparison of ~ 1.2 kb amplicon of R16F2n/R2 primed 16S rDNA sequences of twenty-eight chickpea isolates (Table 2), sesamum isolate (Acc. No. MT420677), six weed isolates (Acc. Nos. MN551490-91, MN551498-9, MT420678-9) and two leafhopper species (Acc. Nos. MT500682-83) showed 98.47% to 100% sequence identity among each other and 100% identity with sunflower phyllody (Acc. No. MK421430), faba bean phyllody (Acc. No. MK453522), sesame phyllody (Acc. No. KF322278), soybean witches’ broom phytoplasma (Acc. No. HQ840717) and other peanut witches’ broom related strains related to 16SrII group.

The *rp* gene sequences of twenty-eight chickpea phytoplasma isolates (Table 2), sesamum isolate (Acc. No. MT423344), six weed isolates (Acc. Nos. MN728266-69, MT423345-46) and two leafhopper species (Acc. Nos. MT501706-07) showed 99.92% to 100% sequence identity with carrot witches’ broom phytoplasma (Acc. No. MH816949), alfalfa witches broom phytoplasma (Acc. No. EF193371), cotton phyllody (Acc. No. EF186814) and crotalaria phyllody (Acc. No. EF186818) strains related to 16SrII group.

Comparison of ~ 840 bp partial sequences of *sec*A gene of twenty-eight chickpea phytoplasma isolates (Table 2), sesame isolate (Acc. No. MT423365), weed isolates (Acc. Nos. MN728248-51, MT423366-67) and two leafhopper species (Acc. Nos. MT501708-09) showed 99.63% to 99.83% sequence identity with tomato big bud phytoplasma (Acc. No. MG251644), carrot phyllody phytoplasma (Acc. No. KX358580) and *Citrus aurantifolia* phytoplasma (Acc. No. KX358586) strains of peanut witches’ broom (16SrII) group.

Comparison of 1700 bp complete sequences of *sec*Y genes of twenty-eight phytoplasma isolates (Table 2), sesamum isolate (Acc. No. MT423386) weed isolates (Acc. Nos. MN728230-33, MT423387-88) and two leaf hopper species (Acc. Nos. MT501710-11) had 98.09% to 99.94% sequence identity with cauliflower phyllody (Acc. No. KC953012), tomato big bud (Acc. No. KT970081), cotton phyllody (Acc. No. GU004350) and crotalaria phyllody (Acc. No. GU004349) phytoplasma strains of 16SrII group.

Also, the *tuf* gene partial 1094 bp of twenty-eight phytoplasma isolates (Table 2), sesamum (Acc. No. MT423407), weed isolates (Acc. Nos. MN634234-37, MT423408-09) and two leafhopper species (Acc. Nos. MT501712-13) had 97.85% to 100% sequence identity with tomato big bud (Acc. No. KX358596), pea phyllody (KX358595), faba bean phyllody (Acc. No. KX358594), carrot phyllody (Acc. No. KX358592) and crotalaria witches’ broom phytoplasma (Acc. No. KY872724) strains identified in 16SrII group.

The complete *imp* gene sequences of twenty-eight phytoplasma isolates (Acc. Nos. MN634204-215, MT423412-27, Acc. Nos. MN634220-21, MN634221, MT423431-32; Table 2), sesamum isolate (Acc. No. MT423428), weed isolates (Acc. Nos. MN634216-19, MT423429-30) and two leafhopper species (Acc. Nos. MT501704-05) revealed 99.42% to 100% sequence homology with periwinkle phyllody (Acc. No. MK453513), cucumber phyllody (Acc. No. MK453510), alfalfa witches’ broom (Acc. No. JQ745274) and 99.42% with faba bean phyllody (Acc. No. JQ745278) phytoplasma strains in 16SrII group.

#### Phylogenetic relationship

**Fig. 5:**
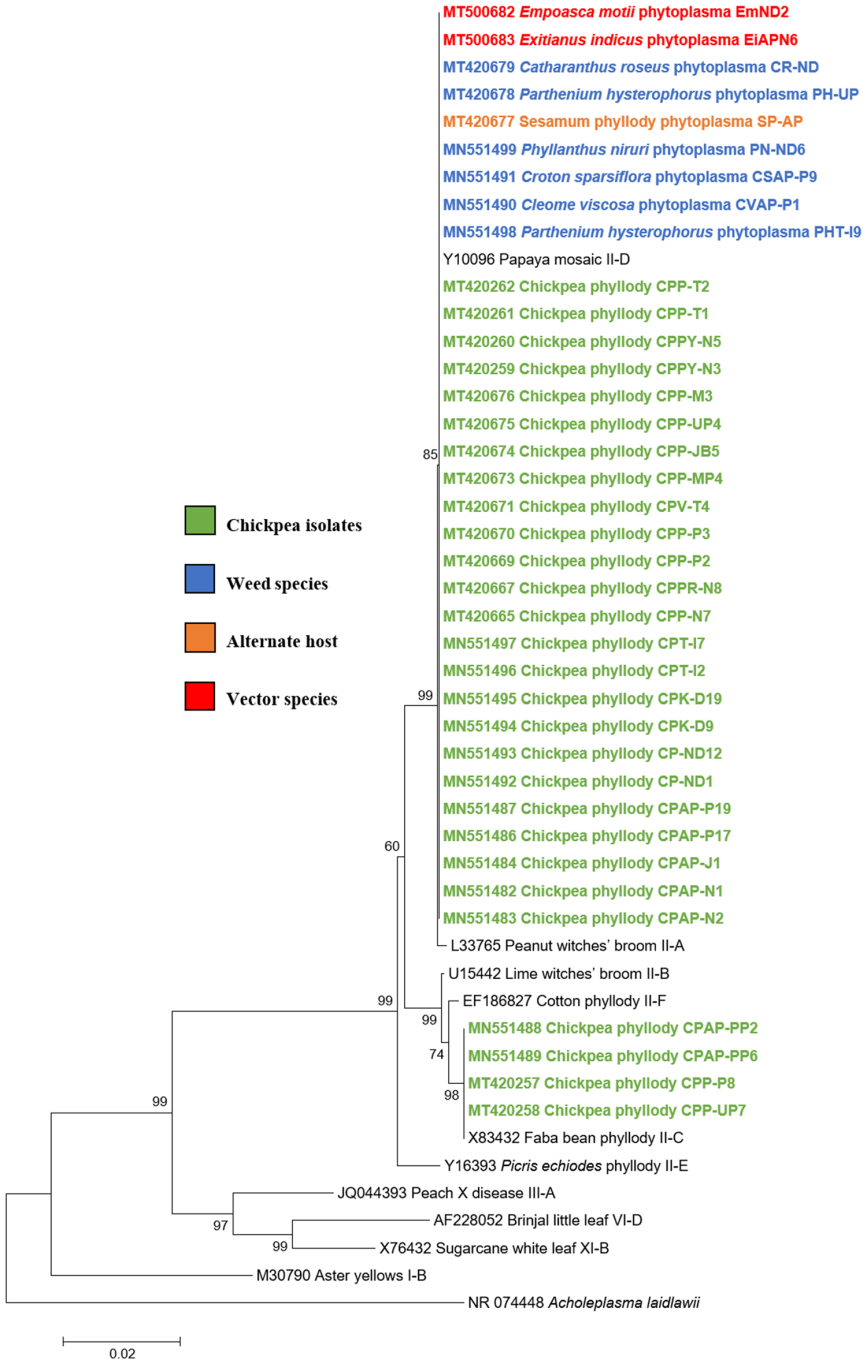
Phylogenetic tree of 16S rRNA gene sequences constructed by neighbor-joining method and Kimura’s three-parameter model, showing the relationships among chickpea phytoplasma isolates, weed isolates and alternate host with reference phytoplasma strains. The tree was rooted with *Acholeplasma laidlawii*. Numbers on branches are bootstrap values obtained for 1000 bootstrap replicates. The bar represents a phylogenetic distance of 0.02

Phylogenetic analysis of the 16S rRNA sequences of twenty-eight chickpea phytoplasma isolates with those of submitted sequences in GenBank revealed their close phylogenetic relationship with members of peanut witches’ broom (16SrII) group. It is evident from the results that twenty-four chickpea isolates, sesamum isolate, two leafhopper species and all the six weed isolates were clustered in subclade with 16SrII phytoplasma group-related strains of 16SrII-D subgroup. However, remaining four (three chickpea phytoplasma isolates from AP and one isolated from UP) were clustered with the phytoplasma strains of 16SrII-C subgroup in phylogeny tree (Fig. 5).

**Fig. 6:**
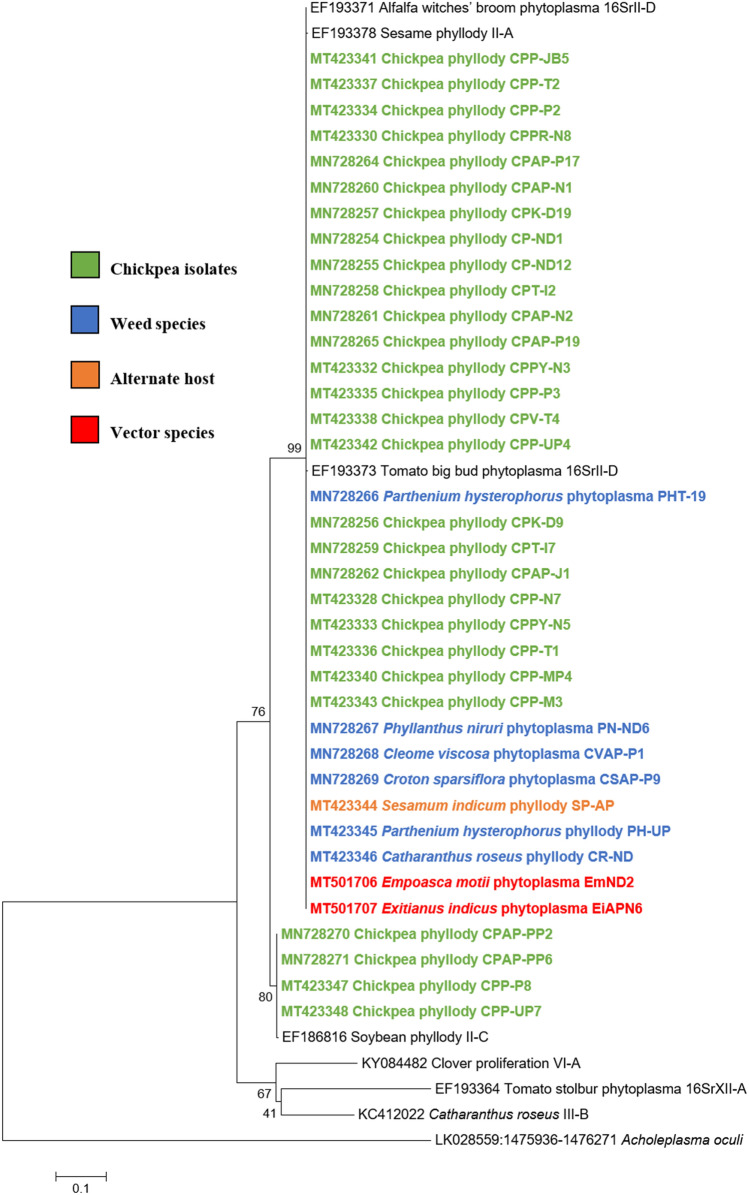
Phylogenetic tree of *rp* gene sequences constructed by neighbor-joining method and Kimura’s three-parameter model, showing the relationships among chickpea phytoplasma isolates, weed isolates and alternate host with reference phytoplasma strains. The tree was rooted with *Acholeplasma oculi*. Numbers on branches are bootstrap values obtained for 1000 bootstrap replicates. The bar represents a phylogenetic distance of 0.1

**Fig. 7:**
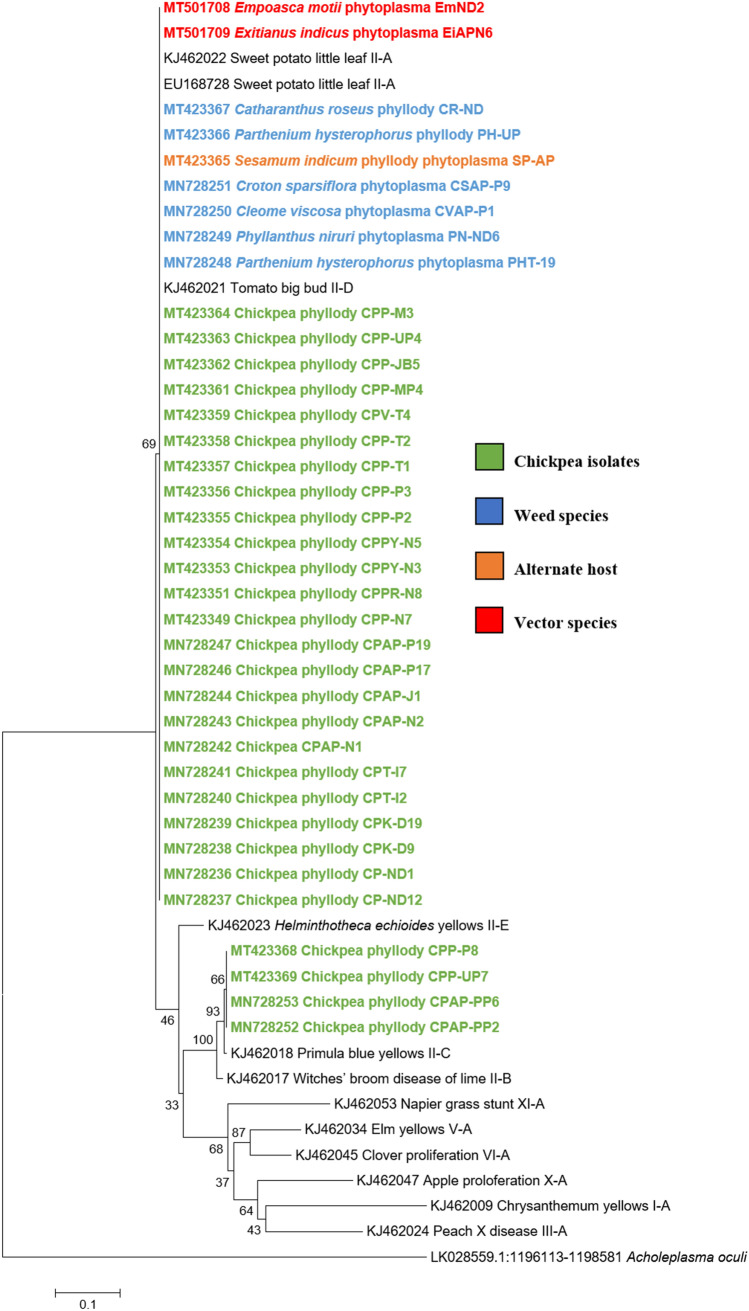
Phylogenetic tree of *sec*A gene sequences constructed by neighbor-joining method and Kimura’s three-parameter model, showing the relationships among chickpea phytoplasma isolates, weed isolates and alternate host with reference phytoplasma strains. The tree was rooted with *Acholeplasma oculi*. Numbers on branches are bootstrap values obtained for 1000 bootstrap replicates. The bar represents a phylogenetic distance of 0.1

**Fig. 8:**
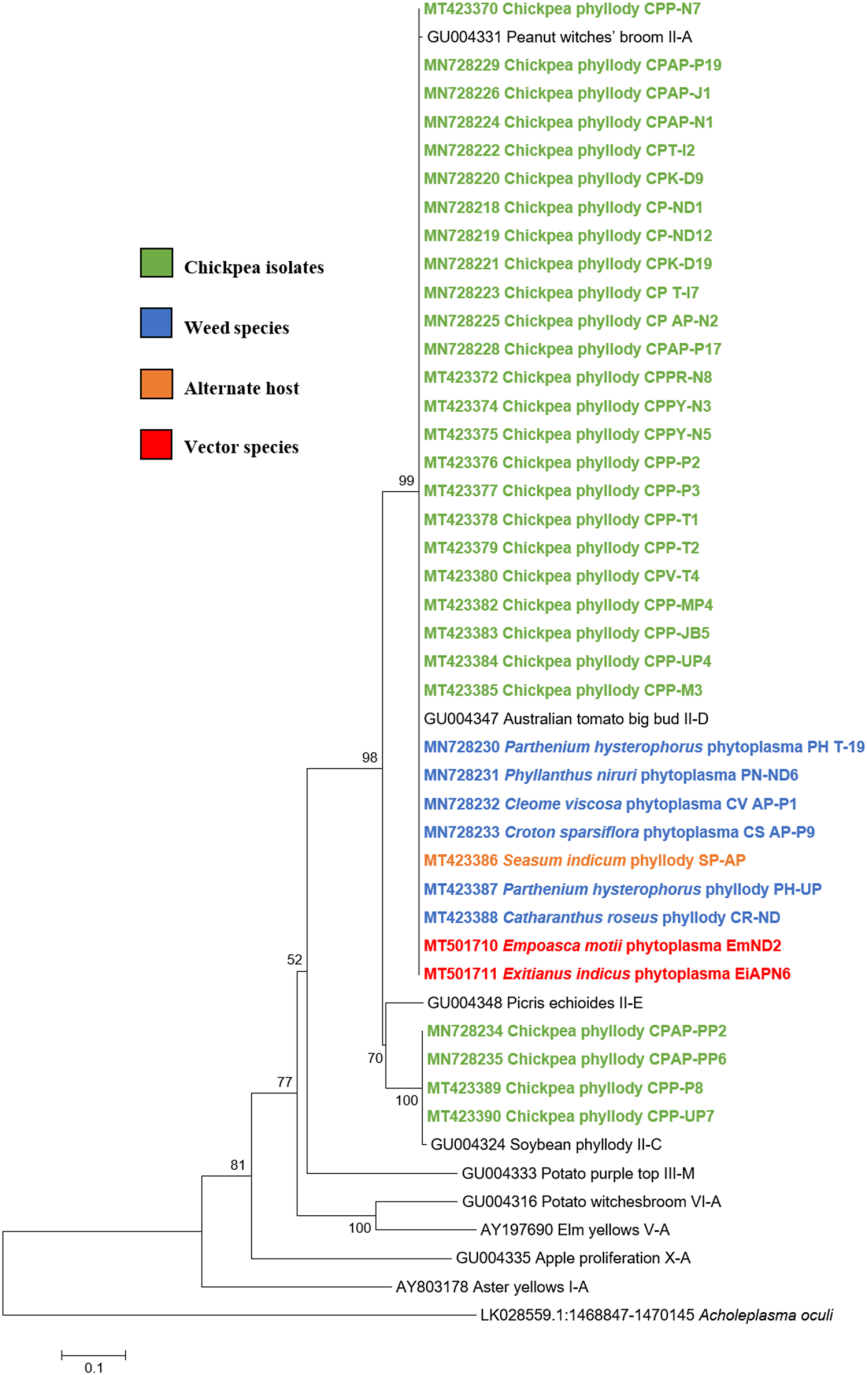
Phylogenetic tree of *sec*Y gene sequences constructed by neighbor-joining method and Kimura’s three-parameter model, showing the relationships among chickpea phytoplasma isolates, weed isolates and alternate host with reference phytoplasma strains. The tree was rooted with *Acholeplasma oculi*. Numbers on branches are bootstrap values obtained for 1000 bootstrap replicates. The bar represents a phylogenetic distance of 0.1

**Fig. 9:**
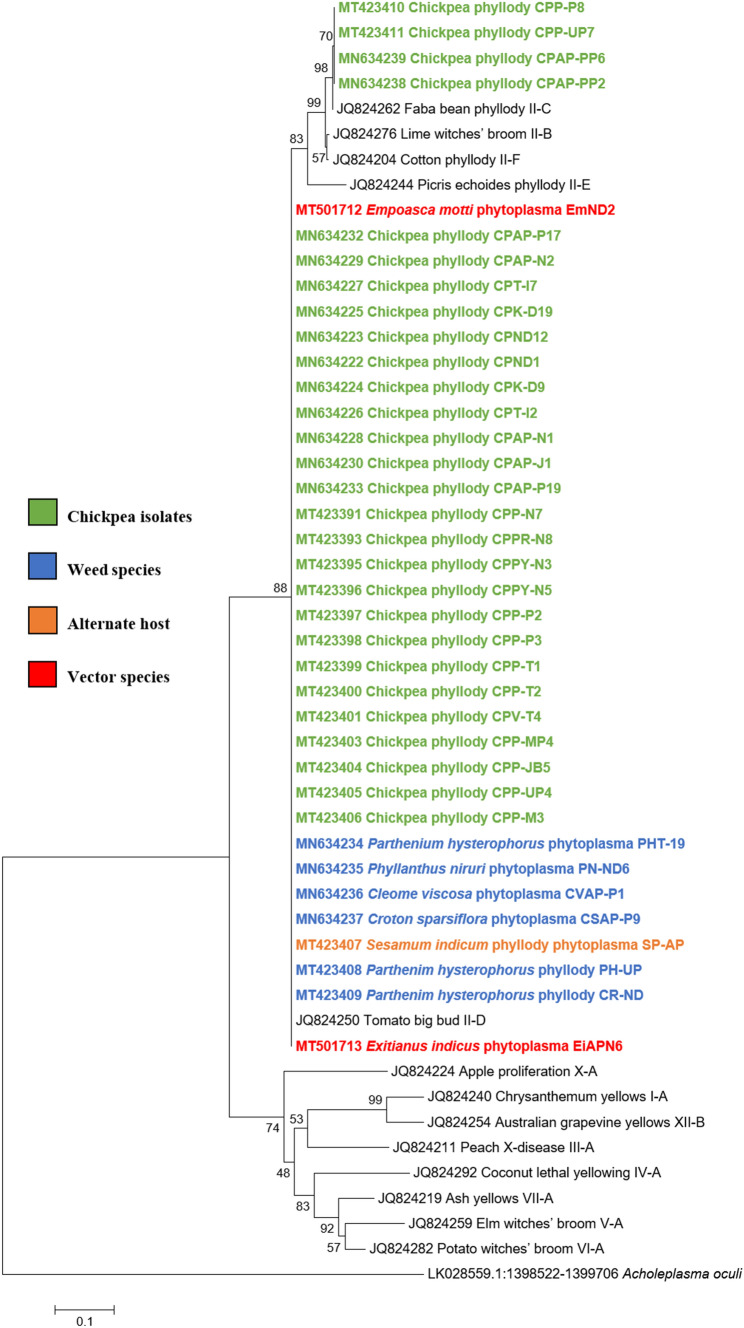
Phylogenetic tree of *tuf* gene sequences constructed by neighbor-joining method and Kimura’s three-parameter model, showing the relationships among chickpea phytoplasma isolates, weed isolates and alternate host with reference phytoplasma strains. The tree was rooted with *Acholeplasma oculi.* Numbers on branches are bootstrap values obtained for 1000 bootstrap replicates. The bar represents a phylogenetic distance of 0.1

**Fig. 10:**
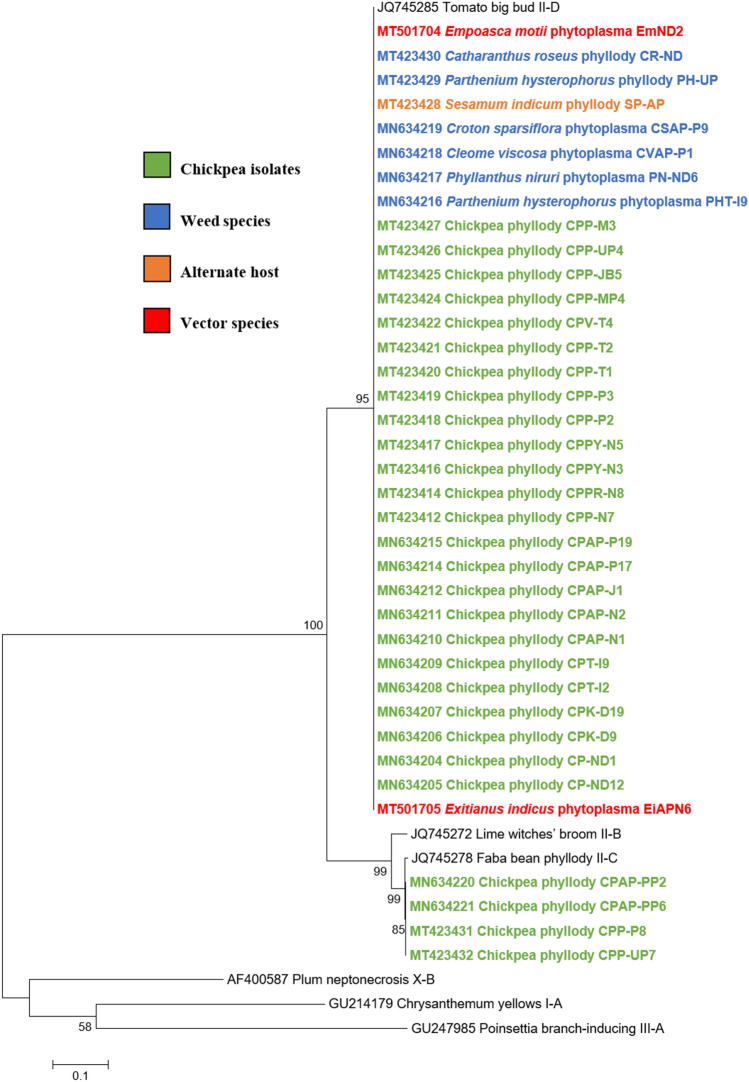
Phylogenetic tree of *imp* gene sequences constructed by neighbor-joining method and Kimura’s three-parameter model, showing the relationships among chickpea phytoplasma isolates, weed isolates and alternate host with reference phytoplasma strains. Numbers on branches are bootstrap values obtained for 1000 bootstrap replicates. The bar represents a phylogenetic distance of 0.1

Similar results were obtained with the phylogenetic comparison analysis with *rp*, *sec*A, *sec*Y, *tuf* and *imp* gene sequences of chickpea phytoplasma isolates when compared with those of reference strains of phytoplasma sequences in GenBank (Figs.  6, 7, 8, 9, 10). The phylogenetic analysis of 16S rRNA, *rp*, *sec*A, *sec*Y, *tuf* and *imp* gene sequences confirmed the association of peanut witches’ broom (16SrII) group with symptomatic chickpea samples in the present study.

The phylogenetic analysis results of 16S rDNA and other multilocus genes (*rp*, *sec*A, *sec*Y, *tuf* and *imp*) sequences of phytoplasma isolates from other host (*S. indicum*), five weed species (*P. hysterophorus, C. viscosa, C. sparsiflora, C. roseus and P. niruri*) and two leafhopper species (*E. indicus* and *E. motti*) also confirmed the similar findings as of chickpea isolates and as they were closely clustered with phytoplasma strains of 16SrII group.

#### In silico RFLP analysis

**Fig. 11:**
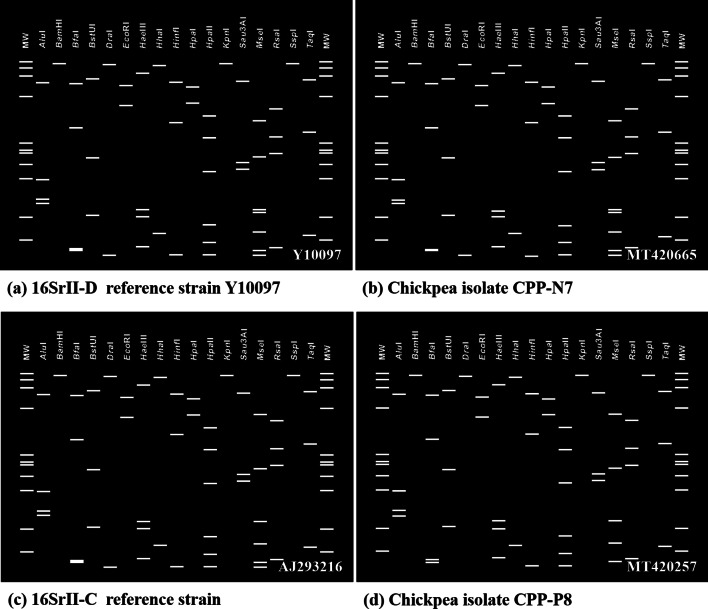
Comparison of virtual RFLP pattern derived from in silico digestion of ~ 1.25 kb 16SrRNA sequences of reference phytoplasma subgroup with 17 different restriction endonucleases using *i*phyclassifier programme **a** 16SrII-D reference strain (Acc. No. Y10097), **b** chickpea isolate CPP-N7 (Acc. No. MT420665), **c** 16SrII-C reference strain (Acc. No. AJ293216), **d** chickpea isolate CPP-P8 (Acc. No. MT420257)

The virtual RFLP analysis of the F2nR2 region of 16S rRNA gene chickpea phytoplasma isolates was compared for the 16Sr group and subgroup assignment using *iPhy*Classifier online tool. Comparison of the restriction site maps revealed that twenty-four isolates (Table 2) produced similar virtual RFLP profile identical to reference strain for 16SrII-D subgroup (Acc. No. Y10097) (Fig. 11 a, b) with the similarity coefficient value of 1.00. However, other three chickpea isolates from AP (Acc. Nos. MN551488, MN551489, MT420257) and one isolate from UP (Acc. No. MT420258) generated restriction patterns identical to that of reference phytoplasma strain, 16SrII-C subgroup (Acc. No. AJ293216) with similarity coefficient of 1.00 (Fig. 11 c, d). On the basis of similar restriction profiles, the chickpea phytoplasma isolates in the present study were classified under peanut witches’ broom group as 16SrII-C and 16SrII-D subgroups-related strains.

The virtual RFLP profiles of the six positive phytoplasma isolates from weeds (Acc. Nos. MN551490-91, MN551498-9, MT420678-9), sesame (Acc. No. MT423386) and two leafhopper species (*E. indicus* and *E. motti,* Acc. Nos. MT500682-83) were found identical with the reference strain of 16SrII-D (Y10097).

We also recorded mixed infection of mastrevirus and phytoplasma (16SrII-D) in four chickpea samples (three isolates from AP and one isolate from Telangana, Table 2).

## Discussion

Chickpea is a leading leguminous crop grown in India, Australia, Pakistan, Myanmar, Turkey and Iran (Anonymous, 2019). In India, it is cultivated across the country as a major summer crop. India has the highest acreage, but the productivity is very low due to the prevalence of biotic and abiotic stresses (Singh et al. 1993). Chickpea stunt (CpS) is a re-emerging disease in all chickpea growing areas of Indian subcontinent, Australia, South Africa and Canada. Four different group of viruses (mastrevirus, luteovirus, polerovirus and cucumovirus) have been reported associated with CpS disease worldwide (Kanakala and Kuria 2019). Chickpea phyllody caused by phytoplasma is another emerging problem of chickpea in some major chickpea growing countries (Shreenath et al. 2020).

Severe growth reduction in chickpea plants caused by BLRV was first reported by Kaiser (1972), and it was named as chickpea stunt by Nene and Reddy (1976). SCRLV and BWYV in California (Bosque-Perez and Buddenhagen 1990; Horn et al. 1993) and BLRV and BWYV in Spain (Carazo et al. 1993) were later identified associated with the disease. A new CpCSV strain of the genus *Polerovirus* was identified with CpSD in Ethiopia causing yellowing and stunting symptoms (Abraham et al. 2006). Later on, a geminivirus was also reported associated with CpSD and it was shown to be transmitted by a leafhopper, *O. albicinctus* from India and Pakistan (Horn et al. 1993; Akhtar et al. 2011). In a recent study, mixed infection of mastrevirus, cucumovirus and phytoplasma was reported associated with CpSD in India (Shreenath et al. 2020).

Besides polerovirus, luteovirus and begomovirus, CMV is also reported to be associated with little leaf, chlorosis and stunt symptoms in chickpea (Chalam 1986; Shreenath et al. 2020). Shreenath et al. (2020) recently identified association of CMV in chickpea stunt plants along with mixed infection of mastrevirus and phytoplasma at IARI, New Delhi. However, no confirmation of individual or mixed infection of CMV was confirmed with symptomatic chickpea samples from any state of India in the present study. The earlier identification of CMV with chickpea stunt samples at IARI may be due to availability of potential natural plant and weed reservoirs being grown in the vicinity of chickpea fields and dominance of efficient aphid vector, *M. persicae* (Shreenath et al. 2020). But the report of association of CMV with CpSD is alarming and may become a serious problem for chickpea cultivation under suitable conditions of availability of favorable plant hosts and insect vectors, which needs future survey and investigation.

The virus indexing of thirty-seven chickpea stunt samples from eight states of India in present study suggested that CpCDV (mastrevirus) is the major cause of CpSD and is widespread in all chickpea growing states of India. CpCDV has a wide host range worldwide including vegetables, pepper, watermelon, cotton, papaya, legumes, tobacco, sesame, mustard and weeds like *Sesbania bispinosa* and *Xanthium strumarium*. (Kanakala and Kuria 2019). In the present study, three new crops (*B. nigra*, *B. juncea* and *L. culinaris*) and two weed species (*H. contartus, A. virginica*) were identified as additional hosts of CpCDV in India. Earlier, CpCDV infection was reported in lentil from Pakistan (Kraberger et al. 2013) and mustard from Australia (Schwinghamer et al. 2010). The presence of CpCDV in lentil and mustard in the present study is the new reports from India. Our study also suggested role of two positive weeds species (*H. contartus, A. virginica*) growing in and around chickpea fields as a putative natural host reservoir of CpCDV and is the new host records in world.

Horn et al. (1993) successfully transmitted CpCDV to different species of leguminous, solanaceous and chenopodiaceous hosts through a leafhopper vector, *O. orientalis*. Akhtar et al. (2011) demonstrated that CpCDV is successfully transmitted by *O. albicinctus* in Pakistan*.* In this study, CpCDV was identified in two species of leafhoppers, *Amarasca biguttula* and *O. albicinctus* feeding in chickpea fields suggesting that these leafhopper species may be potential source of natural vectors of CpCDV infection. The detection of CpCDV in *A. biguttula* is a new report as it may become a potential vector in transmitting CpCDV in new areas under chickpea cultivation in India.

Phytoplasma association with chickpea phyllody disease was first time reported from Coimbatore, India, and phytoplasma association was confirmed on the basis of Diene’s staining (Venkataraman 1959). Till date, the association of phytoplasma with chickpea is reported from Australia, Ethiopia, Oman, Myanmar, Pakistan and Sudan (Akhtar et al. 2008; Al-Saady et al. 2006; Reddy et al. 1991; Saqib et al. 2005). Afterwards, the disease was reported from several major chickpea growing areas in India: Haryana (Sangwan et al. 1981), Karnataka (Pallavi et al. 2012), UP, Maharashtra and Karnataka (Akram et al. 2016), AP (Naik et al. 2018) and New Delhi (Shreenath et al. 2020). In the present study, association of two subgroups of phytoplasma was reported with chickpea stunt and shoot proliferation disease from AP, Telangana, Karnataka, MP, UP and New Delhi. Stunting was a common symptom induced by virus or phytoplasma. But phytoplasma also induced bushy appearance, proliferation of axillary shoots, little leaf and phyllody (Saqib et al. 2005; Pallavi et al. 2012; Shreenath et al. 2020), and in the present study, association of two subgroups of phytoplasma (16SrII-C and 16SrII-D) was identified and characterized with chickpea samples collected from AP, Telangana, Karnataka, MP, UP and New Delhi. Out of twenty-eight chickpea samples, twenty-four chickpea isolates were identified to be associated with strain of 16SrII-D subgroup phytoplasma-related strains, whereas four chickpea samples were associated with 16SrII-C subgroup. Earlier, only phytoplasma strains belonging to 16SrII-D were reported in chickpea from India (Pallavi et al. 2012; Shreenath et al. 2020) and Pakistan (Akhtar et al. 2009). Hence, the report of association of 16SrII-C phytoplasma subgroup with chickpea phyllody disease in the present study is a new report. We also observed stunting and leaf reddening symptoms along with phyllody and witches’ broom in the same chickpea plants from AP and Telangana and detected a mixed infection of CpCDV and 16SrII-D phytoplasma (data not shown).

As the 16S rRNA gene is inadequate for finer differentiation of closely related but distinct phytoplasmas strains, four multilocus genes as *sec*A, *rp*, *sec*Y, *imp* and *tuf* also confirmed and validated for identification of phytoplasma strain in symptomatic chickpea, other crops, chickpea and leafhopper. Our results confirmed the validity and utility of all these multilocus genes as additional suitable molecular markers for authentic characterization of phytoplasma strains belonging to 16SrII-C and 16SrII-D subgroups in all the symptomatic tested plant and insect samples.

In this study, five weed species, viz. *C. viscosa*, *C. sparsiflora* (Andhra Pradesh), *P. hysterophorus* (Telangana and UP), *P. niruri*, *C. roseus* (New Delhi), and one cultivated crop, viz. *S. indicum* (AP), were identified and characterized as hosts for 16SrII-D subgroup of phytoplasmas. All of these weed species except *C. sparsiflora* have been reported earlier as hosts of different phytoplasma groups (Rao et al. 2017). *C. bonplandianum* has been reported as host of 16SrII and 16SrVI-D subgroup of phytoplasmas in India (Kirdat et al. 2020), and we reported another species, *C. sparsiflora* as new host record of 16SrII-D phytoplasma subgroup, which is a new report in world. In the present study, sesamum plants grown as intercrop in chickpea fields in Kadapa district of AP was identified as natural alternate host for 16SrII-D phytoplasma subgroup. Sesame crop has already been reported earlier as host of different groups of phytoplasmas (16SrI, II and VI) in India and abroad (Rao et al. 2015). The sesame reported as host of 16SrII-D subgroup phytoplasma in the vicinity of chickpea fields in AP in the present study may pose a serious threat in spread of chickpea phyllody disease in other chickpea growing regions.

Phytoplasmas are mostly transmitted by sap sucking leafhoppers, planthoppers and psyllids (Weintraub and Beanland 2006; Weintraub et al. 2019). Earlier, *O. orientalis was* identified as the main leafhopper vector for natural transmission of chickpea phyllody phytoplasma in India and Pakistan (Ghanekar et al. 1988; Akhtar et al. 2009; Pallavi et al. 2012). In the present study, two more leafhopper species (*E. indicus, E. motti*) feeding on symptomatic chickpea plants from AP and New Delhi were found positive for the presence of 16SrII-D subgroup strain of phytoplasma. *E. indicus* has been reported as putative vector for phytoplasma associated with sugarcane grassy shoot belonging to 16SrXI group in India (Rao et al. 2014) and *E. motti* for 16SrII-D subgroup in cluster bean and sesame phyllody in India (Rao et al. 2019). The information on additional hosts and insect vectors which are harboring 16SrII-D subgroup phytoplasmas would be important to monitor the weeds and leafhopper population in and around chickpea fields towards proper management of CpSD.

Since the chickpea is a major crop and is being grown in all parts of the country, the reported new putative alternate/collateral hosts and natural leafhopper/planthopper vectors of two phytoplasma strains will facilitate transmission of phytoplasma strains associated with chickpea and other crops in the country. Further studies on screening of chickpea genotypes for resistance, management of insect vectors and alternate/collateral host would be, therefore, essential for developing management strategy of the disease and should be introduced in chickpea varietal development programme.

Our results provide the strong evidence for the genetic diversity of CpCDV and phytoplasma strains association with the CpSD. However, its efficiency of insect vectors involved in natural transmission of virus/phytoplasma strains needs to be investigated in different parts of India. Since symptomatic new weed hosts have been identified for CpCDV and phytoplasma, examining weeds as alternative/collateral host is also necessary in designing efficient management strategies. We have provided evidence for the two phytoplasma strains (16SrII-C and II-D) and CpCDV is currently associated with stunt disease in major chickpea growing states of India. These results reinforce the need to develop innovative management strategies.

## Data Availability

The data that support the findings of this study are available from the corresponding author upon reasonable request.

## References

[CR1] Abraham AD, Menzel W, Lesemann DE, Varrelmann M, Vetten HJ (2006). Chickpea chlorotic stunt virus: a new polerovirus infecting cool-season food legumes in Ethiopia. Phytopathology.

[CR2] Akhtar KP, Ahmad M, Shah TM, Atta BM (2011). Transmission of chickpea chlorotic dwarf virus in chickpea by the leafhopper *Orosius albicinctus* (Distant) in Pakistan-short communication. Plant Prot Sci.

[CR3] Akhtar KP, Shah TM, Atta BM, Dickinson M, Hodgetts J, Khan RA (2009). Symptomatology, etiology and transmission of chickpea phyllody disease in Pakistan. J Plant Pathol.

[CR4] Akhtar KP, Shah TM, Atta BM, Dickinson M, Jamil FF, Haq MA (2008). Natural occurrence of phytoplasma associated with chickpea phyllody disease in Pakistan-a new record. Plant Pathol.

[CR5] Akram M, Sachan DK, Patil DK, Chavan RA, Sontakke PL, Saifulla M (2016). Characterization of phytoplasma associated with chickpea phyllody based on 16S rRNA gene sequence analysis. J Food Legumes.

[CR6] Al-Saady NA, Al-Subhi AM, Al-Nabhani A, Khan AJ (2006). First report of a group 16SrII phytoplasma infecting chickpea in Oman. Pl Dis.

[CR7] Bekele B, Abeysinghe S, Hoat TX, Hodgetts J, Dickinson M (2011). Development of specific *sec*A-based diagnostics for the 16SrXI and 16SrXIV phytoplasmas of the Gramineae. Bull Insectol.

[CR8] Bosque-Perez NA, Buddenhagen IW (1990). Studies on epidemiology of virus disease of chickpea in California. Pl Dis.

[CR9] Carazo G, DeBlas C, Saiz M, Romero J, Castro S (1993). Virus diseases of chickpea in Spain. Pl Dis.

[CR10] Chalam TV, Reddy MV, Nene YL, Subbayya J (1986). Some properties of a strain of cucumber mosaic virus isolated from chickpea in India. Pl Dis.

[CR11] Deng S, Hiruki C (1991). Amplification of 16S rRNA genes from culturable and non-culturable mollicutes. J Microbiol Methods.

[CR12] Folmer O, Black M, Hoeh W, Lutz R, Vrijenhoek R (1994). DNA primers for amplification of mitochondrial cytochrome c oxidase subunit I from diverse metazoan invertebrates. Mol Mar Biol Biotechnol.

[CR13] Ghanekar AM, Manohar SK, Reddy SV, Nene YL (1988). Association of a mycoplasma-like organism with chickpea phyllody. Indian Phytopathol.

[CR14] Gundersen DE, Lee IM (1996). Ultrasensitive detection of phytoplasmas by nested-PCR assays using two universal primer pairs. Phytopathol Mediterr.

[CR15] Hodgetts J, Boonham N, Mumford R, Harrison N, Dickinson M (2008). Phytoplasma phylogenetics based on analysis of secA and 23S rRNA gene sequences for improved resolution of candidate species of ‘*Candidatus* Phytoplasma’. Int J Syst Evol Microbiol.

[CR16] Horn NM, Reddy SV, Roberts IM, Reddy DVR (1993). Chickpea chlorotic dwarf virus, a new leafhopper-transmitted geminivirus of chickpea in India. Ann Appl Biol.

[CR17] Horn NM, Reddy SV, Van den Heuvel JFJM, Reddy DVR (1996). Survey of chickpea (*Cicer arietinum* L.) for chickpea stunt disease and associated viruses in India and Pakistan. Pl Dis.

[CR18] Kaiser WJ (1972). Diseases of food legumes caused by pea leaf roll virus in Iran. FAO Pl Prot Bull.

[CR19] Kanakala S, Kuria P (2019). Chickpea chlorotic dwarf virus: an emerging monopartite dicot infecting Mastrevirus. Viruses.

[CR20] Kanakala S, Sakhare A, Verma HN, Malathi VG (2013). Infectivity and the phylogenetic relationship of a mastrevirus causing chickpea stunt disease in India. Eur J Plant Pathol.

[CR21] Kandaswamy TK, Natarajan C (1974). A note on phyllody disease on Bengal gram (*Cicer arietinum* L.). Madras Agric J.

[CR22] Kirdat K, Tiwarekar B, Thorat V, Narawade N, Dhotre D, Sathe S (2020). Draft genome sequences of two phytoplasma strains associated with sugarcane grassy shoot (SCGS) and bermuda grass white leaf (BGWL) diseases. Mol Plant Microbe Interact.

[CR23] Kraberger S, Harkins GW, Kumari SG, Thomas JE, Schwinghamer MW, Sharman M (2013). Evidence that dicot-infecting mastreviruses are particularly prone to inter-species recombination and have likely been circulating in Australia for longer than in Africa and the Middle East. Virology.

[CR24] Kumar S, Stecher G, Tamura K (2016). MEGA7: molecular evolutionary genetics analysis version 7.0 for bigger datasets. Mol Biol Evol.

[CR25] Kumari SG, Makkouk KM, Attar N, Ghulam W, Lesemann DE (2004). First report of chickpea chlorotic dwarf virus infecting spring chickpea in Syria. Pl Dis.

[CR26] Lee IM, Bottner-Parker KD, Zhao Y, Davis RE, Harrison NA (2010). Phylogenetic analysis and delineation of phytoplasmas based on secY gene sequences. Int J Syst Evol Microbiol.

[CR27] Makkouk KM, Rizkallah L, Kumari SG, Zaki M, Enein RA (2003). First record of chickpea chlorotic dwarf virus (CpCDV) affecting faba bean (*Vicia faba*) crops in Egypt. Pl Pathol.

[CR28] Malathi VG, Kanakala S, Mandal B, Rao GP, Baranwal VK, Jain RK (2017). Diversity and pathogenesis of mastreviruses in India. A century of plant virology in India.

[CR29] Martini M, Lee I, Zhao Y, Botti S, Bertaccini A, Carraro L (2004). Ribosomal protein gene-based phylogeny: a basis for phytoplasma classification. Int Organiz Mycoplasmol.

[CR30] Merga B, Haji J (2019). Economic importance of chickpea: production, value, and world trade. Cogent Food Agric.

[CR31] Naik DVK, Reddy BB, Rani JS, Devi RSJ, Prasad KH (2018). Natural occurrence of phytoplasma associated with chickpea phyllody in Andhra Pradesh, India. Int J Curr Microbiol Appli Sci.

[CR32] Nene YL, Reddy MV (1976). Preliminary information on chickpea stunt. Trop Grain Legume Bull.

[CR33] Nene YL, Reddy MV (1987) Chickpea diseases and their control. Chickpea Diseases and Their Control 233–270

[CR34] Ortiz V, Castro S, Romero J (2005). Optimization of RT–PCR for the detection of bean leaf roll virus in plant hosts and insect vectors. J Phytopathol.

[CR35] Pallavi MS, Ramappa HK, Shankarappa KS, Rangaswamy KT, Wickramaarachchi WART, Maruthi MN (2012). Detection and molecular characterization of phytoplasma associated with chickpea phyllody disease in south India. Phytoparasitica.

[CR36] Rao GP, Tiwari AK, Kuma BVK (2014). Identification of sugarcane grassy shoot-associated phytoplasma and one of its putative vectors in India. Phytoparasitica.

[CR37] Rao GP, Nabi SU, Madhupriya (2015). Overview on a century progress in research on sesame phyllody disease. Phytopathogenic Mollicutes.

[CR38] Rao GP, Panda P, Reddy MG, Mishra S (2019). Identification and management of 16SrII-D phytoplasmas in cluster bean and sesame crops in the Haryana province of India. Phytopathogenic Mollicutes.

[CR39] Rao GP, Thorat V, Manimekalai R, Tiwari AK, Yadav A (2017). A century progress of research on phytoplasma diseases in India. Phytopathogenic Mollicutes.

[CR40] Reddy MV, Baw UA, Hein UM, Moe UK, Su UT, Sethi SC (1991). Survey of chickpea diseases in Myanmar. Intl Chickpea Newsl.

[CR41] Reddy MV, Nene YL, Verma JP (1979). Pea leaf roll virus causes chickpea stunt. Intl Chickpea Newsl.

[CR42] Robertson NL, French R, Gray S (1991). Use of group-specific primers and the polymerase chain reaction for the detection and identification of luteoviruses. J Gen Virol.

[CR43] Sangwan MS, Khirbat SK, Jalali BL (1981). Record of chickpea phyllody [*Cicer arietinum* L.]. Jour Res-Haryana Agric Univ.

[CR44] Saqib M, Bayliss KL, Dell B, Hardf GS, Jones MGK (2005). First record of a phytoplasma-associated disease of chickpea (*Cicer arietinum*) in Australia. Aus Pl Pathol.

[CR45] Schneider B, Seemüeller E, Smart CD, Kirkpatrick BC, Razin S, Tully JG (1995). Phylogenetic classification of plant pathogenic mycoplasma-like organisms or phytoplasmas. Molecular and diagnostic procedures in mycoplasmology.

[CR46] Schwinghamer MW, Thomas JE, Schilg MA, Parry JN, Dann EK, Moore KJ (2010). Mastreviruses in chickpea (*Cicer arietinum*) and other dicotyledonous crops and weeds in Queensland and Northern New South Wales, Australia. Aus Pl Pathol.

[CR47] Singh KB, Malhotra RS, Halila MH, Knights EJ, Verma MM (1993). Current status and future strategy in breeding chickpea for resistance to biotic and abiotic stresses. Euphytica.

[CR48] Shreenath YS, Saritha RK, Basavaraj YB, Pant RP, Sagar D, Arya M (2020). Evidence for the association of mastrevirus, cucumovirus and phytoplasma with chickpea stunt disease and their putative insect vectors in India. Eur J Pl Pathol.

[CR49] Venkataraman K (1959). Phyllody in Bengal gram (*Cicer arietinum* I). Madras Agric J.

[CR50] Weintraub PG, Beanland L (2006). Insect vectors of phytoplasmas. Annu Rev Entomol.

[CR51] Weintraub PG, Trivellone V, Krüger K, Bertaccini A, Weintraub P, Rao GP, Mori N (2019). The Biology and Ecology of Leafhopper Transmission of Phytoplasmas. Phytoplasmas: plant pathogenic bacteria-II, transmission and management of phytoplasma associated diseases.

[CR52] Zhao Y, Wei W, Lee M, Shao J, Suo X, Davis RE (2009). Construction of an interactive online phytoplasma classification tool, *i*PhyClassifier, and its application in analysis of the peach X-disease phytoplasma group (16SrIII). Int J Syst Evol Microbiol.

